# Using social media and personality traits to assess software developers’ emotional polarity

**DOI:** 10.7717/peerj-cs.1498

**Published:** 2023-09-27

**Authors:** Leo Silva, Marília Gurgel de Castro, Miriam Bernardino Silva, Milena Santos, Uirá Kulesza, Margarida Lima, Henrique Madeira

**Affiliations:** 1 Centre of Informatics and Systems, University of Coimbra, Coimbra, Portugal; 2 Faculty of Psychology and Educational Sciences, University of Coimbra, Coimbra, Portugal; 3 Department of Informatics and Applied Mathematics, Federal University of Rio Grande do Norte, Natal, Rio Grande do Norte, Brazil

**Keywords:** Software engineering, Lexicon, Social media, Sentiment analysis, Personality trait, Big Five

## Abstract

Although human factors (*e.g*., cognitive functions, behaviors and skills, human error models, *etc*.) are key elements to improve software development productivity and quality, the role of software developers’ emotions and their personality traits in software engineering still needs to be studied. A major difficulty is in assessing developers’ emotions, leading to the classic problem of having difficulties understanding what cannot be easily measured. Existing approaches to infer emotions, such as facial expressions, self-assessed surveys, and biometric sensors, imply considerable intrusiveness on developers and tend to be used only during normal working periods. This article proposes to assess the feasibility of using social media posts (*e.g*., developers’ posts on Twitter) to accurately determine the polarity of emotions of software developers over extended periods in a non-intrusive manner, allowing the identification of potentially abnormal periods of negative or positive sentiments of developers that may affect software development productivity or software quality. Our results suggested that Twitter data can serve as a valid source for accurately inferring the polarity of emotions. We evaluated 31 combinations of unsupervised lexicon-based techniques using a dataset with 79,029 public posts from Twitter from sixteen software developers, achieving a macro F1-Score of 0.745 and 76.8% of accuracy with the ensemble comprised of SentiStrength, Sentilex-PT, and LIWC2015_PT lexicons. Among other results, we found a statistically significant difference in tweets’ polarities posted during working and non-working periods for 31.25% of the participants, suggesting that emotional polarity monitoring outside working hours could also be relevant. We also assessed the Big Five personality traits of the developers and preliminarily used them to ponder the polarities inferences. In this context, *Openness*, *Conscientiousness*, and *Extraversion* were frequently related to neutral and positive posts, while *Neuroticism* is associated with negative posts. Our results show that the proposed approach is accurate enough to constitute a simple and non-intrusive alternative to existing methods. Tools using this approach can be applied in real software development environments to support software team workers in making decisions to improve the software development process.

## Introduction

The study of the role of emotions in work and professional contexts is a multidisciplinary endeavor involving organizational psychologists, process management, and field domain specialists. From the organizational psychology perspective, emotional and affective issues have received significant attention. They are often linked to behavior in organizational settings (Ashkanasy & Dorris, 2017), where the understanding of the factors shaping individual and corporate performance are of utmost importance (Stets & Turner, 2014).

Many research studies have established different facets of the impact of human factors (*e.g*., sentiments, moods, emotions, and personality traits) on professional work in general (Weiss & Cropanzano, 1996) and on people’s performance at work (Amabile et al., 2005). For instance, Frost (Glasø & Vie, 2009) suggests that unhappy employees tend to be disconnected from their work, which can lead to low productivity and low quality of work. Diener, Thapa & Tay (2020) found that positive emotions influence key variables for workplace success, including creativity (Kaufmann, 2003), reasoning (Chang & Wilson, 2004), memory tasks (Lewis & Critchley, 2003), information processing (Armitage, Conner & Norman, 1999), behavior (Kirchsteiger, Rigotti & Rustichini, 2006), learning (Weiss, 2000), cognitive processing (Rusting, 1998), decision-making (Kirchsteiger, Rigotti & Rustichini, 2006), and performance (Lane et al., 2005).

Software development is a human-intensive intellectual activity where human factors play a major role (Novielli, Calefato & Lanubile, 2015). Modern software development approaches rely on social and communicative processes (Storey, 2012), especially in the context of large-scale software projects, where human factors play a key role (Feldt et al., 2010). The demanding nature, high complexity, and time pressure of software development can lead to frustration and other negative emotions in developers. Monitoring these emotions becomes crucial for enhancing the effectiveness of the development process.

The approaches used so far to assess (*i.e*., to monitor) emotions along large periods include self-assessed surveys (Graziotin, Wang & Abrahamsson, 2013), facial expressions analysis (Kolakowska et al., 2013), and sensors attached to the user’s body (Aranha, Correa & Nunes, 2019), among others. These monitoring approaches have been proposed in research works to investigate the role of emotions and how they may affect the developers’ productivity (Wrobel, 2013; Graziotin, Wang & Abrahamsson, 2014a, 2014b; Girardi et al., 2021; Meyer et al., 2021) and the quality of the software products (Khan, Brinkman & Hierons, 2011; Destefanis et al., 2016; Graziotin et al., 2018; Khan & Saleh, 2021). However, all these approaches imply some degree of intrusiveness and disturbance in normal activities. Although these methods are acceptable for research contexts, their inherent intrusiveness is an obstacle to their acceptance in professional software development environments.

The explosion of social media offers a relevant source of information to monitor emotions in a non-intrusive way, opening the possibility of porting emotion monitoring from research to real-world software development. Social media is a consolidated technology where a massive amount of data is publicly available daily, such as text statements, likes, emoticons, and multimedia attachments.

The objective of this article is to assess the feasibility of using social media posts to accurately determine the polarity of emotions of software developers over extended periods in a non-intrusive manner. The work is focused on evaluating the feasibility of this approach and, more specifically, on evaluating its accuracy.

Our work takes advantage of sentiment analysis technique to categorize each post based on its emotional polarity as either emotionally loaded (positive or negative) or neutral. Emotional polarity analysis aims to identify the primary emotional orientation or sentiment of textual expressions. Within our specific focus on social media posts, we analyze and evaluate emotional polarity to uncover the predominant emotional tone associated with these posts.

Social media encompasses various platforms used for different purposes, including content communities like YouTube and Instagram, social networking sites like Facebook and LinkedIn, and micro-blogging platforms like Twitter and Tumblr. In this article, we used Twitter as posts’ data source because it is the predominant social media platform for gathering user opinions, popularly and widely adopted in research on sentiment analysis (Drus & Khalid, 2019). Twitter enables users to post and engage with concise messages, making it a platform for expressing opinions and, thus, providing valuable information to researchers, businesses, and even government entities.

The use of sentiment analysis on social media has been a topic of research for some time (Drus & Khalid, 2019), however, the true challenge is determining the accuracy of sentiment polarity in social media data. Of the various techniques for assessing sentiment polarity in text data (Serrano-Guerrero et al., 2015), unsupervised lexicon-based methods are particularly noteworthy (Khoo & Johnkhan, 2018). These methods are simple to implement, do not require labeled data, and rely on dictionaries of words with pre-assigned sentiment scores that can be easily updated with domain-specific words. This flexibility makes unsupervised lexicon-based methods a promising option for future use in software development environments. Thus, we formulate our main research question as follows:

*RQ1: Is Twitter data a valid source for inferring the polarity of emotions accurately?*

Performing sentiment polarity classification using social media as a data source is a big challenge. An evident problem is that developers may present a socially desirable image by posting content on social media that does not reflect their true feelings, or even attempt to present a false persona instead of being authentic. Although this is certainly a reality in social media, it is quite challenging to impersonate a character and consistently pretend to be somebody else for a long time. In the long run, people will behave on social media with traces that can reflect their own real-world behavior (Karanatsiou et al., 2022). In this context, software developers tend to maintain an expected behavior based on their personality traits, and social media posts should reflect this behavior (Chen, Magdy & Wolters, 2020).

The correlation between personality traits and emotions polarity classification could indicate that the person’s personality traits may weight emotions in posts written on social media. We used correlation matrices to measure the strength of correlations between developers’ sentiment polarities provided by lexicons and developers’ personality traits. Thus, understanding these correlations may provide additional insights into interpreting lexicons’ polarity classification of tweets. Therefore, we formulated a secondary research question as follows:

*RQ2: Can personality traits of software developers be used to weight the polarities inferred from Twitter posts?*

The need of non-intrusive methods to assess developers’ emotional polarity over long periods is essential to cover the duration of large-scale software projects that could extend for many months or years. In the context of this article, intrusiveness refers to any form of disruption, interference, or deviation from professional activities caused by the mechanism used to determine sentiment polarity. Intrusive methods often disturb developers’ work activities, leading to less engagement and unreliable or missing responses in surveys. The use of social media as a data source, as proposed in this article, is non-intrusive and, importantly, is not limited to working hours. Therefore, we can determine if the sentiment analysis polarity results obtained outside of working hours significantly differ from those obtained during working hours, raising another secondary research question as follows:

*RQ3: Are tweets posted outside working hours different, in terms of polarity, from tweets posted during working hours?*

To investigate our research questions, we analyzed a total of 79,029 tweets in Portuguese from 16 Brazilian software developers over 36 months. To establish a ground truth, a team of psychologists manually classified the polarity of a sample of the posts. We then performed an automated sentiment analysis on the entire dataset using five different lexicon-based methods. Additionally, we examined the potential influence of the participants’ Big Five personality traits (McCrae & John, 1992) on their social media activities.

This article has the following contributions:

- Proposes a new approach to evaluate workers’ sentiment polarity during extended periods without interfering with professional tasks, opening the possibility of monitoring emotions polarity in real-world professional environments;
- Evaluates the proposed approach using software developers as a case study since the software industry is a relevant activity sector that relies on complex human activities that are highly dependent on developers’ emotions;
- Considers different possibilities for implementing the proposed approach by evaluating the accuracy of different unsupervised sentiment analysis methods to classify the sentiment polarity of software developers’ posts on Twitter. The classification obtained by the sentiment analysis methods was compared with the manual classification of a sample of posts evaluated manually by a team of expert psychologists (used as reference) and showed that the best methods could classify the polarity of posts with a macro F1-Score of $0.745$ and an accuracy of $0.768$;
- Benchmarks five lexicon-based sentiment analysis methods and ensembles in a total of 31 combinations of methods and identifies and ranks the best methods and ensembles, guiding the best alternative to assess emotions of software developers using non-intrusive data analysis from social media;
- Shows that developers’ posts polarity on Twitter during and outside working periods could change substantially for some developers, demonstrating that the proposed approach of assessing developers’ emotions from social media data is not only non-intrusive but also covers the entire period of working and non-working time;
- Proposes the use of the Big Five dimensions of personality as a factor to weight the tweets polarities assessed by lexicon-based sentiment analysis methods to support the identification of abnormal negative or positive periods that may influence software development;
- Makes available for public access the anonymized dataset used in this study, including answers to surveys, ethics committee documents, the results of analysis, all generated charts, and data analysis in the companion data (available at https://doi.org/10.5281/zenodo.7846996).

The article is organized as follows: the next section presents the background of relevant topics and discusses the possibilities and challenges of using psychological information in software development. After that, we describe the proposed approach and implementation details. Then, we show the performed experimental study to evaluate how accurately developers’ emotions are inferred from social media, followed by a section that discusses the results. Finally, we present threats to validity, and conclusions.

## Background and state of the art

### Sentiment and emotions

Sentiments and emotions have been used interchangeably (Munezero et al., 2014), mainly because both sentiments and emotions refer to “experiences that result from the combined influences of the biological, the cognitive, and the social” (Stets, 2006).

Emotions are psychological states raised by external or internal stimuli (Plutchik & Kellerman, 1980) or, more broadly, can be related to someone or something (Frijda, 1993). While external stimuli or interpersonal interactions can trigger emotions, they can also arise from internal processes such as thoughts, memories, or physiological changes. It is important to note that emotions can be experienced and felt internally without being specifically directed toward a particular person or object.

Psychologists have proposed many theories and models to classify human emotions (Ortony & Turner, 1990). We can mention The Plutchik Wheel (Plutchik & Kellerman, 1980) and the different basic emotions sets, such as those proposed by Ekman & Friesen (1971) and by Cowen & Keltner (2017). For instance, when a person is experiencing a positive or negative occurrence in his life, watching a dramatic movie or listening to a lovely song, it is not difficult to perceive the emotion in that person.

Cattell (1940) defines sentiment as “an acquired and relatively permanent major neuropsychic disposition to react emotionally, cognitively, and conatively toward a certain object (or situation) in a certain stable fashion, with awareness of the object and the manner of reacting.” Murray & Morgan (1945) defined sentiment as “a more or less enduring disposition (predilection or readiness) in a personality to respond with a positive or negative affect to a specified entity”.

Sentiments differ from emotions by the duration of the experience. Sentiments are constructed and directed toward an object, which is not always true for emotions. Emotions can or not be targeted toward an object. For instance, one person can wake up depressed or happy for no apparent reason (Russell & Barrett, 1999). In this work, we deal with emotions in a broad way. We perform sentiment analysis to identify whether a social media post is emotionally loaded (*positive* or *negative*) or not (*neutral*).

### Personality traits

Psychologists consider *personality* as a person’s unique long-term pattern of thinking, emotions, and behavior. Psychology also characterizes personalities in terms of traits, which are relatively stable characteristics that influence person’s behavior across many situations. It also refers to the particular varieties of talents, values, hopes, loves, hates, and habits that make each person unique (Mischel, 2004).

There are several models and theories to classify personality traits, such as Personality Types (Jung, Baynes & Beebe, 2016) (understandable through the MBTI (Myers, 1962) indicator). In this work, we use the Five-Factor model (known as Big Five) (McCrae & John, 1992) (also mentioned by the acronym OCEAN), one of the most accepted and used models to trace personality traits (Barroso et al., 2017; Lin, Mao & Zeng, 2017; da Silva & Paraboni, 2018; Aqeel Iqbal, Aldaihani & Shah, 2019; Karanatsiou et al., 2022). According to McCrae & John (1992), most human personality traits can be reduced to five large dimensions despite language or culture. A person could score low or high on each dimension or factor.

The Big Five personality traits consist of five general factors: *Openness*, *Conscientiousness*, *Extraversion*, *Agreeableness*, and *Neuroticism*. *Openness* is associated with a person’s intellect and imagination, reflecting their creativity and willingness to explore new things. *Conscientiousness* is the desire to pursue goals and complete tasks accurately. *Extraversion* measures a person’s openness to external interactions *vs* a preference for solitude. *Agreeableness* indicates social harmony, while *Neuroticism* refers to an individual’s tendency to experience negative emotions like anxiety and anger.

Previous research has shown that certain personality traits are associated with specific emotional experiences and how personality traits regulate emotions (Hughes et al., 2020). For instance, individuals who score high on *Neuroticism* tend to experience negative emotions more frequently, trying to reduce them immediately (Carver & Connor-Smith, 2010). Those high in *Conscientiousness* are less reactive to negative affect, and those high in *Agreeableness* have a reduced sensitivity to their daily stresses (Komulainen et al., 2014).

### Text sentiment analysis

There are two main techniques for text sentiment analysis tools: unsupervised lexicon-based and supervised machine learning-based.

Machine learning approaches are potentially more effective, but they are also more complex that lexicon-based approaches. Machine learning approaches rely on building a classifier (model) with appropriate feature selection, and training such model with an annotated dataset (Hu & Liu, 2006). Naïve Bayes classifier, support vector machine (SVM), and random forest are well-known models for sentiment classification through machine learning. Since supervised machine learning-based needs a training dataset to design a classifier, the training dataset may be unavailable.

The unsupervised lexicon-based approach uses a sentiment lexicon with words and phrases that are positive and negative (Khoo & Johnkhan, 2018). A list of linguistic features labeled according to their semantic orientation (positive or negative) comprises a sentiment lexicon. Researchers first create a sentiment lexicon by compiling sentiment word lists using manual, linguistic, and *corpus*-based approaches, then determine the polarity score of the given review based on the positive and negative indicators.

Our work involves a large dataset of unlabeled tweets, which makes unsupervised lexicon-based techniques a more viable option than machine learning approaches. This is particularly relevant for future use of the proposed method in real-world software development scenarios. In this article, we evaluate several unsupervised lexicon-based techniques and determine the best alternatives. As the developers who participated in the study are native speakers of Brazilian Portuguese, we used lexicon-based techniques specifically created or adapted for this language. These techniques include:

- *SentiStrength* (Thelwall, Buckley & Paltoglou, 2012): a well-known sentiment analysis method that uses a lexical dictionary labeled by humans enhanced by machine learning. This method used an expanded version of the LIWC dictionary, adding new characteristics for the context of social media;
- *Sentilex-PT* (Carvalho & Silva, 2015): a sentiment lexicon specifically designed for the sentiment and opinion analysis about human entities in texts written in Portuguese, consisting of 7,014 lemmas and 82,347 inflected forms;
- *Linguistic Inquiry and Word Count (LIWC)* (Pennebaker et al., 2015): aims to analyze texts to detect emotional, social, cognitive words and standard linguistic dimensions of texts. Although LIWC has several metrics, we employed only those related to emotional polarities in this study;
- VADER (Hutto & Gilbert, 2014): a gold standard lexicon, and rule-based sentiment analysis tool claimed to perform exceptionally well in a social media context;
- OpLexicon (Souza et al., 2011): a sentiment lexicon for the Portuguese language built using multiple sources of information. The lexicon has around 15,000 polarized words classified by their morphological category, annotated with positive, negative, and neutral polarities.

### State of the art

Research on the psychological aspects and their impact on software development have been growing in recent years. A study from Serebrenik (2017) mentions that emotions expressed by developers are gaining attention within the software engineering research community. In fact, the software development process is highly dominated by human factors. It is technically complex, requires individual demanding cognitive tasks, as well as effective group interaction skills, which justify the growing research focus on the psychological aspects of software development (Graziotin, Wang & Abrahamsson, 2014a).

Several studies have covered sentiment analysis and its relationship with diverse aspects of software development, including issue reopening in software projects (Cheruvelil & Da Silva, 2019), pull-request discussions (Ortu, Marchesi & Tonelli, 2019), issues and tickets (Jurado & Rodriguez, 2015), communication channels (Murgia et al., 2014), code review process (Asri et al., 2019), programming performance (Graziotin, Wang & Abrahamsson, 2015), debugging performance (Khan, Brinkman & Hierons, 2011), social awareness (Calefato & Lanubile, 2012). In addition to this wide range of topics, developers’ sentiment has been particularly researched in the context of software productivity (Wrobel, 2013; Graziotin, Wang & Abrahamsson, 2014a, 2014b; Girardi et al., 2021; Meyer et al., 2021) and software quality (Destefanis et al., 2016; Graziotin et al., 2018; Khan & Saleh, 2021).

Two recent interdisciplinary studies that involve sentiment analysis and software artifacts are worth noting due to its relevance for our study: Girardi et al. (2021) found an association between positive valence and developers’ self-assessed productivity using biometric sensors, and Khan & Saleh (2021) proposed a framework for investigating the impact of emotions on the quality of software artifacts. Both studies found that positive emotions do not always contribute to good-quality artifacts, and negative emotions could sometimes positively impact the quality of artifacts.

Most of these research works have performed sentiment analysis through self-assessed surveys or controlled experiments. Unlike those previous works, this article aims to evaluate the possibility of using social media posts as a data source to assess developers’ emotional polarities in a long-term fashion and without causing any disturbance to the developers.

The use of social media data to perform scientific research is not new. Prior work used information on social media to predict daily ups and downs of the stock market (Bollen, Mao & Zeng, 2011), to predict the political crowd behavior (Makazhanov, Rafiei & Waqar, 2014), and to explain temporal variations in social happiness (Dodds et al., 2011).

In software engineering, researchers have used social media to monitor end-users’ opinions on a given application system (Banerjee et al., 2021) and customer’s perspectives as positive or negative (Sarlan, Nadam & Basri, 2015). Sentiment analysis has also been used to classify tweets as positive or negative after first categorizing them as objective or subjective (Barbosa & Feng, 2010). Twitter has been used to analyze, classify, and interpret emotions that contain software jargon words or software-relevant tweets (Williams & Mahmoud, 2017), and analyze the sentiment of Facebook posts to determine people’s emotions towards an issue (Zamani et al., 2014). A study by Neri et al. (2012) applied sentiment analysis to marketing and proposed sentiment analysis on two broadcasting services through posts on social media.

Another important related area is Affective Computing (AC), which encompasses a wide interdisciplinary research field that studies the development of computational systems able to recognize and react to human emotions (Picard, 2000). Poria et al. (2017) defines AC as a set of techniques that perform affect recognition from data in different modalities and at different granularity scales, enabling intelligent systems to recognize, feel, infer and interpret human emotions. Usually, AC applications use wearable devices, sensors, and webcams as a source of information to identify human emotions and try to adapt software behavior according to the user’s emotional state and content recommendation (Fong et al., 2012).

Although usually used with wearable devices and sensors, AC also works with text data to perform sentiment analysis. The basic tasks of AC and sentiment analysis are emotion recognition and sentiment polarity detection. In this sense, some previous approaches used SenticNet (Cambria et al., 2016), a domain-independent resource for sentiment analysis containing 50,000 commonsense concepts, for tasks such as opinion holder detection (Gangemi, Presutti & Reforgiato Recupero, 2014), knowledge expansion (Wu et al., 2011), subjectivity detection (Chenlo & Losada, 2014), event summarization (Shah et al., 2016), short text message classification (Gezici et al., 2013), sarcasm detection (Poria et al., 2016), Twitter sentiment classification (Bravo-Marquez, Mendoza & Poblete, 2014), deception detection (Jaiswal, Tabibu & Bajpai, 2016), user profiling (Xie et al., 2016), emotion visualization (Scharl et al., 2016), and business intelligence (Egger & Schoder, 2017).

In the software engineering context, Kolakowska et al. (2013) contributed to improving software engineering processes such as extended software usability testing, software quality, and developers’ productivity. A recent study involving emotions and perceived productivity on software development applied biometric sensors through a wristband to perform sensor-based emotion prediction at the workplace (Girardi et al., 2021). These approaches using cameras and biometric sensors, although quite accurate since they provide direct measurements of people’s sentiments, are quite intrusive to the participants and, generally, cannot be applied in real software development environments. Our research, on the contrary, explores a much less intrusive alternative since social media data is already available, and software developers do not need to wear or install any sensors or even feel the pressure of being “observed” by a camera.

Intrusive methods of assessing emotions, such as those mentioned above, offer several advantages. These methods provide direct measurements of people’s emotions, which can be more reliable than other methods, such as observation or inference. Additionally, these methods allow researchers to have greater control over data collection, resulting in increased standardization and consistency across participants. Intrusive methods also enable researchers to study rare or specific emotions, as well as individual differences in emotional experience.

Although virtually non-intrusive, sentiment analysis techniques using information from social media have the open problem of guaranteeing an acceptable level of correctness. In other words, if a post is classified as representing a positive, negative, or neutral sentiment, it is necessary to ensure that such a post effectively represents the classified polarity.

Several evaluation techniques have been proposed, such as manual and automatic polarity labeling of posts and a division of posts into groups of objective and subjective. One of the techniques is performing an annotation process using Shaver framework (Shaver et al., 1987), such as in Murgia et al. (2014), Novielli, Girardi & Lanubile (2018), Calefato et al. (2018a), Novielli et al. (2020), and Herrmann et al. (2022). These evaluation techniques (especially manual post annotation) are often used in supervised learning approaches to train prediction models on manually labeled data (Williams & Mahmoud, 2017).

The need for manual post annotation represents a clear difficulty in applying supervised learning techniques in real data sets from social media. Therefore, an alternative approach is to evaluate the quality of sentiment inferred from social media using unsupervised approaches. Although these techniques are easy to apply in real-world environments, they face the difficulty of collecting and maintaining a universal sentiment lexicon, as different words may reflect different meanings in different contexts (Williams & Mahmoud, 2017). In this work we evaluate the accuracy of lexicon-based techniques as pragmatic and easy to apply alternative to supervised machine learning approaches.

There is a large body of research related to the lexicon-based sentiment analysis for the English language, which is the original language of many lexicons. Since, in our case, the posts on Twitter are written in Brazilian Portuguese, we used unsupervised lexicons designed or adjusted to the Brazilian Portuguese language. We avoided the automatic translation of the posts into English (to allow the use of lexicons designed for English) because, as already discussed, these translation services are unreliable and do not produce good results (Cirqueira et al., 2017). Given that our proposed approach uses unsupervised lexicons designed for the Brazilian Portuguese language, we present relevant previous studies that employed similar methods and utilized Twitter as a data source.

Souza & Vieira (2012) evaluated the lexicons Sentilex-PT and OpLexicon using tweets written in the Brazilian Portuguese language. Their approach obtained the best result of 0.55 F1-Score on classifying positive tweets and 0.45 for negative tweets. As we will present in the following sections, our proposed approach achieved better results in F1-Score for both polarities with an ensemble of lexicons.

The UniLex approach employs Brazilian Portuguese to generate the base of a lexical dictionary (França de Souza, Ramos Pereira & Hasan Dalip, 2017). The authors used a database of 14,084 tweets, mostly related to the political context, to assess the approach. The main results showed a macro F1-Score of 53% and an accuracy of 62%. Like our approach, UniLex uses data originally written in Brazilian Portuguese and does not use any translation mechanisms, reducing the risk of noise from this translation. Our study presents better results using an ensemble of lexicons for accuracy and F1-Score.

Previous work by Cirqueira et al. (2017) attempted to compare sentiment analysis lexicons for English and Brazilian Portuguese by translating social media data from platforms like Facebook and Twitter. However, the translation services used are unreliable, which the authors acknowledged. For instance, the correctness rates of translations ranged from 55% to 57%. The authors employed various sentiment analysis methods, including OpLexicon, VADER, SentiStrength, and SO-CAL. While their best accuracy on Facebook data was 50.60% and on Twitter data was 38.76%, we present in section *Results and Discussion* that our study achieved improved results in accuracy and F1-Score by using an ensemble approach on Twitter data.

In another study by Reis et al. (2015), the authors conducted sentiment analysis on movie and product reviews, tweets, and messages using the iFeel tool. Unlike the previous work discussed, the authors relied solely on lexicons in the original English version, employing automatic translation with Google Translator to analyze posts in various languages. Their findings indicated that the VADER lexicon best predicted positive (F1-Score = 92%) and negative (F1-Score = 87%) polarities in Portuguese. Despite the automatic translation, the authors acknowledged that lexicons in the original English version might be better tuned for the task, which they emphasized in their conclusions.

Previously mentioned studies have utilized Twitter as a data source. While some have reported comparable results to our study’s lexicon analysis, they have not demonstrated quality control to ensure the validity of Twitter as a reliable data source. For example, Akram & Tahir (2019) collected emoticons from Twitter to identify emotions through lexicons, but their study lacked expert evaluation and only focused on emoticons instead of the entire tweet.

A relevant research line proposes methods and tools specifically designed to perform sentiment analysis in the software engineering domain. SentiStrength-SE (Islam & Zibran, 2018) is a domain-specific tool designed for sentiment analysis in technical content, demonstrating better performance than domain-independent techniques. Still, in the software engineering context, Biswas et al. (2020) explored the potential effectiveness of customizing BERT models for sentiment analysis and has also achieved reliable sentiment analysis in software engineering texts. Senti4SD (Calefato et al., 2018a) is also a classifier specifically trained for the software engineering context by supporting sentiment analysis in Stack Overflow questions, answers, and comments, reducing negative bias compared to general sentiment analysis tools.

Previous studies have explored the psychological aspects and their relation to software engineering (Wrobel, 2013; Guzman & Bruegge, 2013; Jongeling, Datta & Serebrenik, 2015; Ortu et al., 2015; Blaz & Becker, 2016; Ortu et al., 2016; Wrobel, 2016; Gachechiladze et al., 2017; Souza & Silva, 2017). However, most of these studies relied on general-purpose sentiment analysis tools trained on non-technical texts. SentiStrength (Thelwall, Buckley & Paltoglou, 2012) has been widely used in software engineering studies, but its accuracy has been questioned for software engineering tasks (Jongeling et al., 2017; Lin et al., 2018). In this context, several customized sentiment analysis tools and methods have been proposed specifically for software engineering, including SentiStrength-SE (Islam & Zibran, 2018), SentiCR (Ahmed et al., 2017), Senti4SD (Calefato et al., 2018a) and EmoTxt (Calefato, Lanubile & Novielli, 2018). In our work, we took a different approach by analyzing posts on an open-context public platform instead of focusing solely on technical environments, allowing our approach to applying to organizations across various work areas and expanding its potential impact beyond software engineering.

Considering the Big Five model in sentiment analysis based on personalities is crucial for real-world applications. Previous studies have attempted to predict Big Five personality dimensions from social media using existing algorithms or dictionaries (Lin, Mao & Zeng, 2017; Tadesse et al., 2018; Darliansyah et al., 2019). However, inferring the dimensions of *Openness* and *Neuroticism* through automated methods presents challenges, and existing studies have reported low accuracy in predicting personality traits (Bai et al., 2013; Adalı & Golbeck, 2014). This challenging prediction holds in software engineering context (Calefato et al., 2018a; Calefato & Lanubile, 2022). The estimated correlation between self-reported and predicted personality traits is generally below 0.5 (Novikov, Mararitsa & Nozdrachev, 2021).

## Proposed approach and implementation details

**Figure 1:**
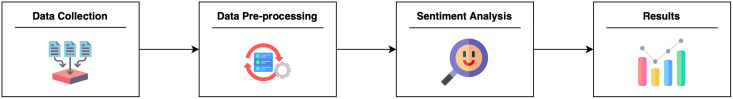
Proposed approach overview.

We used the Python programming language to develop a four-module approach (source code available at https://doi.org/10.5281/zenodo.7844671) for analyzing emotions and sentiments in social media data: data collection, data pre-processing, sentiment analysis, and results. Figure 1 illustrates these modules.

The *data collection* module retrieves tweets from social media platforms, specifically using the Twitter API for extracting Portuguese tweets. To achieve this, we set the API parameter to “pt.” However, the API is limited to retrieving only the 3,200 most recent tweets. To overcome this limitation and obtain a larger number of tweets from a specific user, we employ a web crawler that accesses the user’s Twitter timeline and extracts the required information. It is worth noting that the language of the posts does not play any relevant role in our research, as the results and conclusions are not dependent on the language of the posts.

The collected tweets undergo *pre-processing* to clean and filter the data. The approach removes multimedia attachments, such as images, videos, and animated gifs, as these could interfere with sentiment classification. In addition, the approach removes retweets, as they may not reflect the author’s sentiment, user citations in original tweets, stop words, repeated letters, and URL links. The resulting dataset contains only original and text-based tweets.

After pre-processing, using a lexicon-based technique, our approach performs *sentiment analysis* over tweets. We created a setup that executes lexicons separately and in ensembles. We analyze tweets using the standard method of the lexicons, resulting in a score between −1 and +1. We employed the general normalization function for the scores out of this range. Emoji scores, if present, are also evaluated using the ranking method by Kralj Novak et al. (2015). The final score for each tweet is the average of the text analysis score and the emoji score. We executing ensembles, we determine tweet’s polarity score by taking the average of the scores from each lexicon in the ensemble.

With the calculated polarity scores, the approach categorizes the scores as negative, positive, or neutral using threshold values of −0.05 and +0.05, as prescribed by Hutto & Gilbert (2014). Tweets with scores lower than the negative threshold are *negative*, those with scores higher than the positive threshold are *positive*, and the remainder are *neutral*, keeping the pattern adopted by lexicons and by previous works in sentiment polarity classification (Lin et al., 2018; Novielli, Girardi & Lanubile, 2018; Novielli et al., 2020; Herrmann et al., 2022). In the *results* module, the approach provides the findings from the sentiment analysis.

Considering our goals and research questions, the main advantages of the proposed approach are:

- Our approach is *non-intrusive*. Using social media posts as a data source to assess sentiment polarity does not cause any form of disturbance, interruption, or deviation from the professional activities of software developers. Thus, our approach takes advantage of social media posts without introducing new activities that may disturb professional tasks;
- Our approach allows to *look back in time*. Users posts their social media content deliberately, each at a specific frequency of activity, generating a huge amount of data from the day they register on a social media platform. Thus, we can look back in time at users’ activities, perform sentiment analysis polarity classification and understand their sentiment patterns day-by-day, using this valuable information to suggest software development improvements;
- Our approach is *non-inductive*. We consider social media posts that users have already written or the ones they will ordinarily write. We do not ask for or force any post at a specific time or context. What we get from social media is what users deliberately post;
- We use *consolidated methods*. Our approach employs five traditional lexicon-based methods of sentiment analysis and creates 31 combinations of them to analyze the dataset;
- We use *open-context data*. Despite social media posts might not be, in general, related to software engineering, their polarities affect professional activities (Weiss & Cropanzano, 1996), once these posts’ polarities are a result of what users’ live, face, and how they react. We analyzed users’ open-context posts from an open-context platform and did not apply any restrictions related to the text content or context.

## Experimental study

The next subsections describe the main elements of the experimental study proposed in this article.

### Participants

Sixteen Twitter users have been engaged in the study. After searching in the “Programming (Technology)” topic of Twitter, we privately invited a total of 45 users according to the following criteria:

- Having a profile completely open, with explicit location and direct message enabled;
- Having at least one tweet per day, considering the study period;
- Being a Brazilian software developer that lives in Brazil;
- Having posts mainly in the Brazilian Portuguese language.

We invited 45 developers based on these criteria, obtaining 16 responses. These developers were geographically dispersed across Brazil and worked for different companies. Sixteen developers voluntarily and anonymously agreed to participate in the study. Additional details about participant recruitment and survey guidelines can be found in the Experiment Protocol document, which is available as part of the companion data (available at https://doi.org/10.5281/zenodo.7846996).

The participants were asked to complete a two-part survey: the Demographic Survey and the Big Five Inventory (BFI). The first survey asked them to indicate their Twitter user names and provide demographic information, such as gender, age, schooling, and experience working with software development. The BFI consists of a set of calibrated questions to assess the personality traits of each participant. The psychologists involved in the study analyzed the answers and calculated the scores.

Together with this survey, we asked the participants to read documents related to ethics and privacy. We initially contacted the participants through direct messages on Twitter. Then, we sent an email with the surveys to those who agreed to engage in the study. The participants took both surveys individually. They answered the Demographic Survey on the Google Forms platform. Related to Big Five Inventory, they received a Word document with the 44-item to answer and sent it through email back to researchers.

The characteristics of the participants are presented in Table 1. The majority of the participants were highly educated (14/16) and young adults (9/16) with extensive software development experience (6/16). The data shows more males (62.5%) than females (37.5%) among the participants, indicating gender disparity in the software industry. The bold values highlight the highest values in each category.

**Table 1:** Participants’ characteristics.

		Quantity (%)
Gender	**Male**	**10 (62.5)**
	Female	6 (37.5)
Age	Less than 20	1 (6.25)
	**21–30**	**9 (56.25)**
	31–40	6 (37.5)
Experience in software development	1–3 years	3 (18.8)
	3–5 years	3 (18.8)
	5–7 years	1 (6.3)
	7–10 years	3 (18.8)
	**More than 10 years**	**6 (37.5)**
Schooling	High school	2 (12.5)
	**Higher education**	**14 (87.5)**

**Note:**

The bold values highlight the highest values in each category.

**Figure 2:**
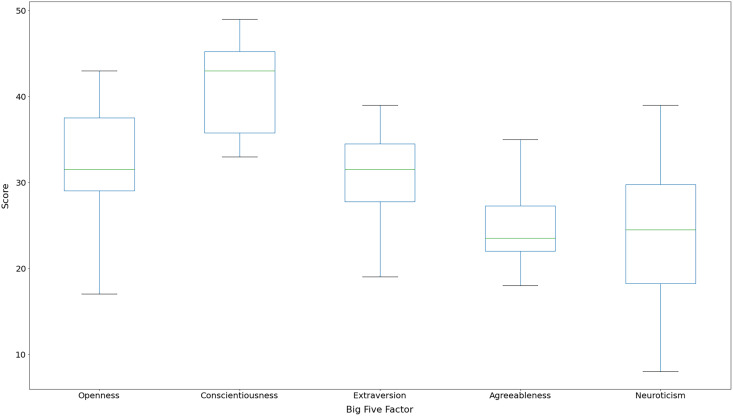
Boxplot for participants’ Big Five factor scores.

Figure 2 presents the boxplots for participants’ Big Five personality traits scores, ranging from 0 to 50. We can note that all participants achieved high scores for *Conscientiousness* (mean = $41.5$; std = $5.29$), indicating that they are highly motivated, disciplined, dedicated, and trustworthy. Previous work has identified that software development teams contain persons with high *Conscientiousness* (Yilmaz et al., 2017). We also found that the scores for *Neuroticism* (mean = $24.19$; std = $8.41$) factor had high dispersion, suggesting that there is no scoring pattern in this factor among the participants. *Agreeableness* (mean = $24.81$; std = $4.86$) was the factor with less dispersion, and half of the scores were considered *medium-low* (median = $23.5$).

### Evaluators

As a key part of our study, we invited three psychologists (co-authors of this article) to collaborate by manually classifying the polarity of a sample of tweets to establish a ground truth. We make available more details general guidelines on surveys in the Experiment Protocol document on the companion data (available at https://doi.org/10.5281/zenodo.7846996).

**Figure 3:**
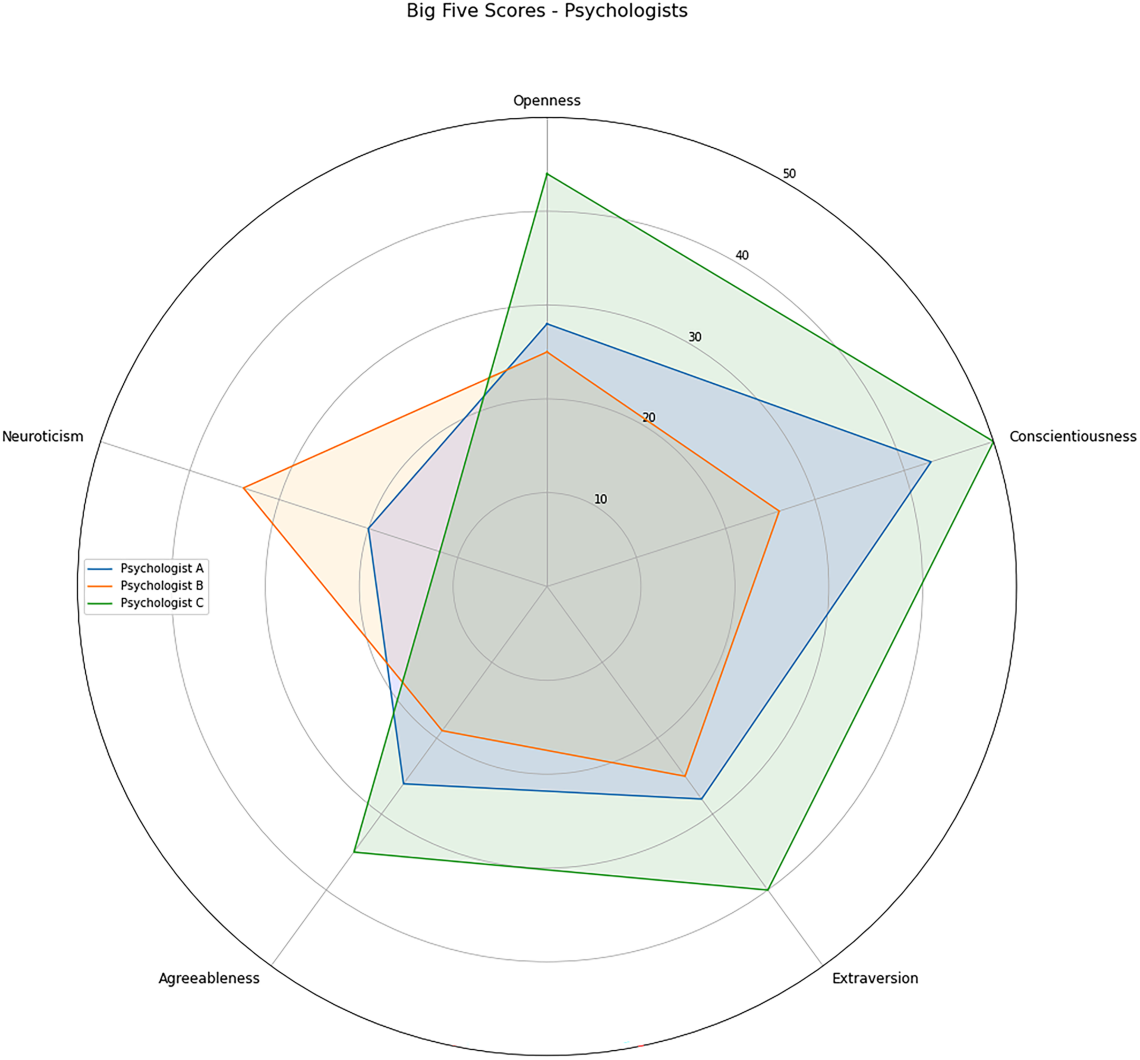
Psychologists Big Five factor personality traits scores.

In the context of this study, the psychologists were called as *evaluators*. To better understand any potential challenges or discrepancies in the manual classification process, we administered the BFI to the evaluators and inferred their personality traits. The results of the BFI are presented in Fig. 3, providing insight into the evaluators’ personality characteristics.

The evaluators have different personality profiles. Psychologist A, represented in blue, scored high on *Conscientiousness*, medium-low on *Neuroticiscm*, and medium on the other three factors. Psychologist B, shown in orange, scored medium-high on *Neuroticism*, medium-low on *Agreeableness*, and medium on other factors. Psychologist C achieved the highest *Conscientiousness* score. This psychologist also scores high on *Extraversion*, *Openness*, medium-high on *Agreeableness*, and medium-low on *Neuroticism*.

### Tweets dataset

This study analyzed Twitter data collected from 16 users over 36 months, from March 2018 to March 2021, resulting in 91,632 tweets (mean = $5,\!727$; std = $5,\!256$). The tweets covered a wide range of topics, and we did not apply any restrictions on the textual content posted by the authors. After pre-processing, the dataset used for analysis contained 79,029 (mean = $4,\!939$; std = $3,\!421$) original text-based tweets. Table 2 shows the number of tweets for each participant, identifying how many were original tweets and how many were retweets.

**Table 2:** The tweets’ dataset.

Participant	Original tweets	Retweets	Total
1	4,569	469	5,038
2	4,376	172	4,548
3	5,828	104	5,932
4	2,704	467	3,171
5	5,417	576	5,993
6	9,193	192	9,385
7	1,433	112	1,545
8	2,210	385	2,595
9	6,451	5	6,456
10	4,558	150	4,708
11	3,393	551	3,944
12	2,065	163	2,228
13	3,985	401	4,386
14	2,459	239	2,698
15	15,481	8,596	24,077
16	4,907	21	4,928
Total	79,029	12,603	91,632
Mean	4,939	788	5,727
Std	3,421	2,090	5,256

When collecting data, we consider a 36-month period for three main reasons. Firstly, as we had a limited number of participants in the study, a longer period allowed us to collect a more substantial tweets dataset. Secondly, the period covered the most challenging phase of the COVID-19 pandemic, which had the potential to impact people’s psychological states. The 36 months period would give us a comprehensive understanding of the typical behavior of participants and allow us to gain a deeper insight into variations in emotional states over time. Thirdly, psychologists consider that a person has a long-term pattern of behavior. Therefore, software developers tend to maintain an expected behavior based on their personality traits, and social media posts should reflect this behavior (Chen, Magdy & Wolters, 2020). Thus, the extended period of 36 months tends to diminish or even mitigate whether a developer tries to impersonate someone else.

### Lexicons

Our study employed the following lexicons: VADER (Hutto & Gilbert, 2014), LIWC2015_PT (Pennebaker et al., 2015), SentiStrength (Thelwall, Buckley & Paltoglou, 2012), OpLexicon (Souza et al., 2011), and Sentilex-PT (Carvalho & Silva, 2015). We chose to use lexicons because our tweets dataset was not labeled. Although supervised machine learning-based methods might have produced more accurate results, they would have required labeling, making their practical use difficult. Therefore, evaluating our proposed approach using lexicons is more relevant and feasible. We executed these lexicons separately and in ensembles.

We use a *post hoc* combination to create 31 ensemble combinations from the five lexicons. We evaluate each ensemble’s performance by comparing it with ground truth data and calculating *precision*, *F1-Score*, and *accuracy* metrics. Each tweet’s polarity score is determined by taking the average scores from each lexicon in the ensemble. We explain this process further on.

After evaluating various lexicons and ensemble combinations and selecting the most optimal one based on the macro F1-Score criteria, we apply the ensemble approach to the entire dataset to obtain the final sentiment classification results. As the macro F1-Score summarizes the overall quality of the classification, we use this metric to leverage the best-performing ensemble combination to provide accurate and reliable sentiment predictions on a large scale.

#### Ethics and privacy

Using social media as a data source may raise participants’ privacy issues, even though this study only uses public posts. Although participants agree with the terms and conditions for Twitter social media, this does not exempt researchers from taking all privacy care in the data analysis (Woodfield, 2017).

We ask participants to read the *Data Protection Statement* (http://bit.ly/3rtzlNI) and to sign the *Informed Consent Statement* (http://bit.ly/3v4nhVh). The former statement informs the participant about what data we need for the study (including social media, Big Five personality traits, and demographic data), how we collect these data, what we will do with the data, and how we guarantee data protection, anonymity, and privacy. The latter statement asks the participant to authorize us to collect these data and perform the study.

Another issue that comes to play is anonymity, especially when involving psychological issues. When working with social media, we must consider anonymity more carefully than traditional research methods. Social media companies store data and metadata for long periods, and much of this data is searchable (Woodfield, 2017). Protecting the participants’ identity is a key issue when dealing with sensitive data. All participants will be treated anonymously in the context of the academic contributions of this study.

We applied the research to the Research Ethics and Deontology Committee of the Faculty of Psychology and Educational Sciences of the University of Coimbra. The committee analyzed and approved our proposal to enhance the software development process through unsupervised lexicon-based sentiment analysis methods. Documents submitted to Ethics Committee are available in the companion data (available at https://doi.org/10.5281/zenodo.7846996).

## Results and discussion

This section discusses the results obtained in our study. The different subsections cover the results of the manual analysis (used as ground truth), the results obtained with the lexicons, the assessment of developers’ emotions, the differences observed in working and non-working periods, the correlation of the results with the personality traits of each participant and the discussion of the possibility of using personality traits to weight the polarity of the tweets measured automatically through lexicon methods.

### Manual analysis

The evaluators manually classified a sample of the tweets to establish the ground truth. This sample was randomly composed of 35 posts for each participant, totalizing 560 anonymous tweets. Each evaluator analyzed this sample separately in a spreadsheet. The goal of this analysis was to classify each tweet regarding its polarity as *negative*, *neutral*, or *positive*, using the following criteria:

- Only tweets with texts were considered;
- Ignore external links;
- Consider tweets’ emotional polarity (positive, negative, or neutral) according to tweets’ writing content;
- Tweets with factual information are considered neutral;
- Classify tweets with mixed sentiments as positive or negative based on the most relevant sentiment;
- Considering positive and negative emphasis, such as emojis, punctuation, and capital letters;

Using our criteria, we requested that the participants categorize their tweets in the same sample. This additional step provided valuable control over the quality of tweets’ classification.

Table 3 shows the overall manual analysis results. The classification was different according to each evaluator, which was somewhat expected, given the clear difference observed in their personality traits (see Fig. 3). For instance, there was a significant difference between classifications for neutral tweets. Psychologist C evaluated 328 posts as neutral, while psychologist B evaluated only 98 in this category. Table 3 also shows that Psychologist A found many positive tweets and an equivalent number of negative and neutral tweets. Psychologist B evaluated more negative tweets among all evaluators but also found a large number of positive tweets. Finally, Psychologist C evaluated fewer negative tweets than others but evaluated more neutral ones. In summary, Psychologist A identified more positive tweets, Psychologist B had the highest number of negative tweets, and Psychologist C had the highest number of neutral tweets. Although we cannot conclude that these differences in the polarity classification directly reflect the personality traits of the evaluators, it is interesting to note that they are reasonably expected considering the dominant personality factors of each evaluator, particularly the factor N (*Neuroticism*).

**Table 3:** Psychologists manual analysis responses.

	Negative	Neutral	Positive
Psych. A	132	126	302
Psych. B	206	98	256
Psych. C	82	328	150

We measured pairwise agreement between the three evaluators with Cohen’s Kappa index and multiple agreements between them with Fleiss’ Kappa index, as shown in Table 4. This table shows that the highest pairwise agreement occurred between Psychologist B and Psychologist C, achieving a Cohen’s index of $0.75$, while Fleiss’ index among all psychologists was $0.64$.

**Table 4:** Pairwise agreement between psychologists with Cohen’s Kappa index and multiple agreement between them with Fleiss’ Kappa index.

Kappa indexes	
Cohen’s Kappa index	
Psychologist A × Psychologist B	0.63300
Psychologist A × Psychologist C	0.55122
Psychologist B × Psychologist C	0.75100
Fleiss’ Kappa index	
All psychologists	0.64523

In Table 5, we can observe the differences in the classification of tweets by the different evaluators. The evaluations were unanimous in 175 (31.25%) tweets and divergent in 385 (68.75%), meaning that at least one evaluator has assigned a classification different from the one assigned by the other evaluator. We used a simple majority to assign the final classification to each tweet, as done in other previous works (Murgia et al., 2014; Blaz & Becker, 2016).

**Table 5:** Results of the manual classification process.

	Tweets’ classification
Unanimous	175 (31.25%)
Divergent	385 (68.75%)
Majority	509 (90.89%)
Completely divergent	51 (9.11%)

The results in Table 5 showed that evaluators successfully classified 509 (90.89%) tweets in one of the three polarity categories by a simple majority. However, the evaluators assigned completely different classifications in 51 (9.11%) tweets, not reaching a final classification. Despite the manual classification criteria presented before, this analysis depends on the evaluator’s perception of what the author was trying to communicate and the polarity embedded in each post. For instance, the evaluators tried to imagine the proposed dialogue between the developer and their followers in a tweet. In addition, the evaluators perceived that language nuances from different regions could affect how tweets were written. Another challenge was due to one of the characteristics of our dataset. In some cases, participants wrote and commented on tweets about software development, which are unfamiliar to the evaluators (psychologists) and may cause classification divergences.

The different personality traits of evaluators were also among the causes of the 51 tweets without a simple majority classification. To overcome the divergences of these 51 tweets, the evaluators performed another round of analysis to converge to a simple majority classification, discussing the main challenges and reasons for the disagreements (Novielli, Girardi & Lanubile, 2018). After this new round, evaluators could reach a classification for all 51 tweets, and they highlighted some difficulties in the divergences, such as tweets containing irony, jokes, complaints, criticism, or praise.

As asked, the participants provided their classification. However, three participants did not label their tweets, resulting in 455 labeled tweets for participants. We then used these 455 manually labeled tweets from participants to compare to evaluators’ ones, thus, considering the latter as ground truth. This step is particularly important because we intend to assess developers’ sentiments using non-intrusive techniques. In fact, asking developers in a real software development environment to label their tweets regarding polarity is intrusive. Thus, to avoid this intrusiveness, it is important that a set of expert professionals precisely perform this labeling step. We will evaluate the lexicons with these results.

Table 6 shows the results. We observed high agreement between the evaluators and participants analysis, with an accuracy of $0.865$ and a macro F1-Score of $0.855$. The results were better for positive classification (precision = $0.926$; recall = $0.863$; F1-Score = $0.893$) than negative (precision = $0.769$; recall = $0.870$; F1-Score = $0.816$). This table also shows the Cohen’s Kappa index between these two classifications: from evaluators and participants. According to Landis & Koch (1977), the Kappa index of $\kappa = 0.710$ indicates a substantial strength of agreement and confirms good reliability of the ground truth.

**Table 6:** Agreement between participants manual analysis and psychologists manual analysis.

	Negative		Positive
Precision	0.76923		0.92638
Recall	0.86957		0.86286
F1-Score	0.81633		0.89349
Accuracy		0.86517	
Macro F1-Score		0.85491	
Cohen’s Kappa index		0.71045	

Previous work (Murgia et al., 2014; Novielli, Girardi & Lanubile, 2018; Calefato et al., 2018a; Novielli et al., 2020; Herrmann et al., 2022) employed an annotation process using Shaver framework (Shaver et al., 1987). These previous works used non-experts as evaluators, such as computer science students (Calefato et al., 2018a; Herrmann et al., 2022), IT professionals (Herrmann et al., 2022). In line with the recommendations of Novielli, Girardi & Lanubile (2018), we provided detailed guidelines for the manual classification of tweets to our evaluators and participants. We achieved a Cohen’s Kappa index ( $\kappa = 0.710$) similar to those achieved by Calefato et al. (2018a) ( $\kappa = 0.740$) and Novielli et al. (2020) ( $\kappa = 0.740$). Differently from previous works, we used experts to perform manual classification and confirmed the reliability of this process with the authors of the tweets.

In the context of this study, we could use either manual classification by the evaluators or the authors to establish a ground truth for the tweet’s polarity. The substantial strength of Cohen’s Kappa index ( $\kappa = 0.710$) indicates that the results would be highly consistent. In this article, we adopted the classification made by experts as the ground truth, which implies that psychologists can label a sample of developer’s tweets with a polarity in a realistic software development environment. Using psychologists results instead of participants contributes to the non-intrusive characteristic of the approach.

It is worth noting that the manual classification of a sample of the posts described in this section was only needed to evaluate the accuracy of the emotion polarity classification obtained with lexicons, which is essential to assess the feasibility of the proposed approach since the polarity of emotions must be evaluated accurately. Obviously, the use of the proposed approach using lexicon in a real software development scenario does not require the manual classification of posts by psychologists or developers.

### Lexicon analysis (RQ1)

Ensemble learning techniques, in general, produce more accurate polarity predictions by combining different classifiers (Ko et al., 2014). Table 7 presents the results of the automated analysis for emotionally loaded posts using each of the lexicons and an ensemble of them. In this table, VA represents the VADER lexicon, OP means OpLexicon, SS indicates SentiStrength, SL means Sentilex-PT, and LI implies LIWC2015_PT. Due to width page restrictions, we also show *Precision* as *Prec*., *F1-Score* as *F1-Sco*., and *Accuracy* as *Acc*.

**Table 7:** Unsupervised lexicon-based analysis metrics for the five sentiment lexicons and ensembles.

Lexicon	Positive			Negative			Acc.	Macro F1-Sco.
	Prec.	Recall	F1-Sco.	Prec.	Recall	F1-Sco.		
VA	0.774	0.722	0.747	0.573	0.638	0.604	0.691	0.675
VA + OP	0.759	0.770	0.765	0.579	0.564	0.571	0.696	0.668
VA + OP + SS	0.779	0.790	0.784	0.594	0.578	0.586	0.717	0.685
VA + OP + SS + SL	0.824	0.791	0.807	0.615	0.663	0.638	0.748	0.723
VA + OP + SS + SL + LI	**0.833**	0.812	0.822	0.636	0.670	0.653	0.765	0.738
VA + OP + SS + LI	0.794	0.818	0.806	0.610	0.574	0.592	0.737	0.699
VA + OP + SL	0.801	0.781	0.791	0.602	0.631	0.616	0.729	0.703
VA + OP + SL + LI	0.812	0.804	0.808	0.620	0.633	0.626	0.747	0.717
VA + OP + LI	0.778	0.794	0.786	0.602	0.579	0.590	0.719	0.688
VA + SS	0.800	0.771	0.785	0.596	0.637	0.616	0.724	0.701
VA + SS + SL	0.802	0.758	0.779	0.610	0.670	0.638	0.726	0.709
VA + SS + SL + LI	0.819	0.782	0.800	0.623	0.676	0.648	0.745	0.724
VA + SS + LI	0.817	0.783	0.800	0.604	0.653	0.627	0.740	0.714
VA + SL	0.801	0.737	0.767	0.605	0.688	0.644	0.719	0.706
VA + SL + LI	0.816	0.763	0.789	0.624	0.695	0.658	0.739	0.723
VA + LI	0.786	0.733	0.759	0.563	0.632	0.596	0.698	0.678
OP	0.719	0.776	0.747	0.514	0.439	0.474	0.658	0.610
OP + SS	0.759	0.824	0.790	0.617	0.521	0.565	0.720	0.678
OP + SS + SL	0.763	0.802	0.782	0.650	0.596	0.622	0.724	0.702
OP + SS + SL + LI	0.792	0.842	0.816	0.682	0.606	0.642	0.757	0.729
OP + SS + LI	0.791	0.838	0.814	0.625	0.549	0.585	0.743	0.699
OP + SL	0.748	0.753	0.751	0.592	0.586	0.589	0.690	0.670
OP + SL + LI	0.776	0.794	0.785	0.615	0.589	0.602	0.721	0.693
OP + LI	0.754	0.808	0.780	0.520	0.442	0.478	0.690	0.629
SS	0.824	0.841	0.833	0.623	0.594	0.608	0.765	0.720
SS + SL	0.790	0.820	0.805	0.696	0.653	0.674	0.756	0.739
SS + SL + LI	0.804	0.838	0.821	**0.698**	0.645	0.670	0.768	**0.745**
SS + LI	0.812	0.857	0.834	0.651	0.573	0.610	0.767	0.722
SL	0.766	0.746	0.756	0.674	**0.698**	**0.686**	0.725	0.721
SL + LI	0.799	0.782	0.790	0.648	0.670	0.659	0.740	0.725
LI	0.825	**0.869**	**0.846**	0.605	0.520	0.559	**0.772**	0.703
Mean	0.791	0.794	0.792	0.615	0.608	0.610	0.730	0.701
Std	0.026	0.036	0.026	0.041	0.065	0.048	0.027	0.030

**Note:**

The best results are shown in bold.

Results on Table 7 show that all lexicons and ensembles performed better on classifying positive than negative tweets (see mean and std in the bottom lines of in Table 7). Previous works also reported the differences between negative and positive performances for Brazilian Portuguese sentiment analysis (Souza & Vieira, 2012; Balage Filho, Pardo & Aluísio, 2013; Cirqueira et al., 2017; Reis et al., 2015).

The best results are spread among several lexicons and ensembles, as observed in the bold values in Table 7. The ensemble of all five lexicons achieved the best precision measure for positive posts, scoring $0.833$. LI scored best recall ( $0.869$) and F1-Score ( $0.846$) for positive posts. For negative tweets, SL (recall of $0.698$; F1-Score of $0.686$) and an ensemble of SS, SL, and LI (precision of $0.698$) concentrates the best results. In general, the LI lexicon provided better quality in inferring positive polarities of tweets. For inferring negative tweets, SL achieved the best results. In both cases, LI and SL achieved the precision metric (for positive and negative tweets, respectively) close to the best value. However, considering the best accuracy and macro F1-Score, LI (accuracy = $0.772$; macro F1-Score = $0.703$) and the ensemble SS + SL + LI (accuracy = $0.768$; macro F1-Score = $0.745$) had the best results.

Our results show that the ensemble comprised of SS + SL + LI performed better on classifying the tweets, reaching a macro F1-Score of 74.5% and an accuracy of 76.8%. This high F1-Score result shows that social media data can be used as a non-intrusive source for sentiment analysis with acceptable accuracy, opening the possibility of researching the polarity of sentiments of software developers as an element to improve the software development process.

Since the ensemble composed by SS + SL + LI provide the best result, we use this ensemble to analyze all tweets in the dataset. The result shows that most participants had a higher percentage of positive than negative tweets. Some individuals, such as P4, P5, P6, P7, P9, and P16, showing a particularly high proportion (Table 8). However, our analysis also showed a balance between negative (26.33%), neutral (34.98%), and positive (38.69%) tweets, indicating that there was no clear pattern in the participants’ social media behavior in general.

**Table 8:** Big Five Factor scores and tweets’ polarities (%) for each participant.

P.	Big Five Factor (Codification)					Tweets (%)		
	O	C	E	A	N	Neg.	Neu.	Pos.
P1	37	43	36	24	26	34.54	37.73	27.73
P2	41	43	34	22	23	26.78	43.74	29.48
P3	43	46	31	24	15	32.19	33.30	34.51
P4	17	33	33	22	39	17.42	27.26	55.33
P5	22	33	31	20	8	23.09	29.98	46.93
P6	39	49	39	28	15	23.07	28.51	48.42
P7	35	35	32	26	16	24.15	31.75	44.10
P8	29	45	34	35	29	25.75	37.56	36.70
P9	40	46	38	22	21	18.63	29.39	51.98
P10	32	46	19	18	28	36.00	29.35	34.64
P11	31	36	25	33	21	21.19	34.69	44.12
P12	36	45	31	31	19	26.00	46.88	27.12
P13	29	41	28	22	28	28.43	41.58	29.99
P14	29	43	27	23	35	31.11	38.55	30.34
P15	29	45	36	27	32	30.22	41.11	28.67
P16	30	35	24	20	32	22.72	28.31	48.97
Mean	32.44	41.5	31.12	24.81	24.19	26.33	34.98	38.69
Std	6.97	5.29	5.41	4.86	8.41	5.43	6.18	9.67

**Note:**

In this table, *O* refers to *Openness*, *C* means *Conscientiousness*, *E* names *Extraversion*, *A* refers to *Agreeableness*, and *N* describes *Neuroticism*.

**Figure 4:**
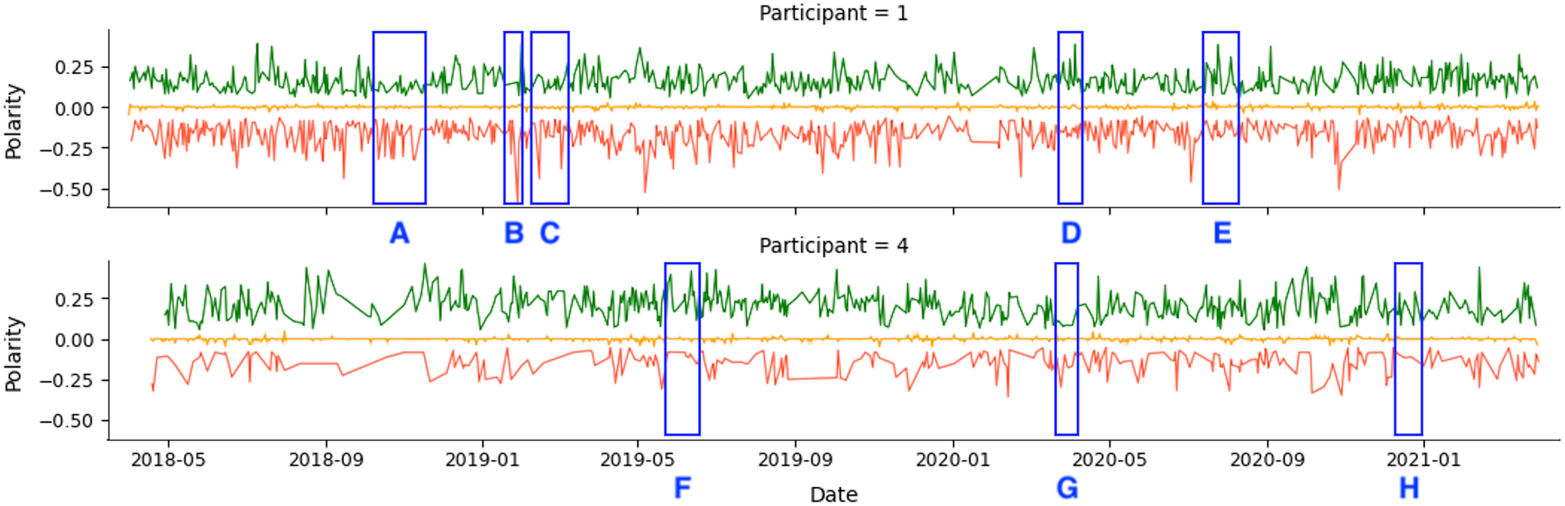
Example of participants’ tweets polarities within the entire research period.

As an example, we show the tweets from two participants over a 36-month period in Fig. 4. The green line represents positive tweets, the orange represents neutral tweets (within the threshold of (−0.05, +0.05)), and the red represents negative tweets. The figure shows variability in negative and positive tweets, with participants showing predominantly negative or positive behavior on certain days or weeks. For instance, Participant 1 (P1) tweets exhibit periods of negativity peak as highlighted in *A*, *B*, and *C*, but also show positive behavior in *D* and *E*. Table 8 confirms that Participant 1 (P1) had more negative (34.54%) than positive (27.73%) tweets.

Participant 4 (P4) had a lower level of social media activity compared to Participant 1 (P1), but the data revealed a higher percentage of positive (55.32%) compared to negative (17.42%) activities on social media. This aligns with the results in Table 8. The regions labeled *F* and *H* indicate days with a higher number of positive tweets. In comparison, region *G* represents a day with a higher number of negative tweets.

**Figure 5:**
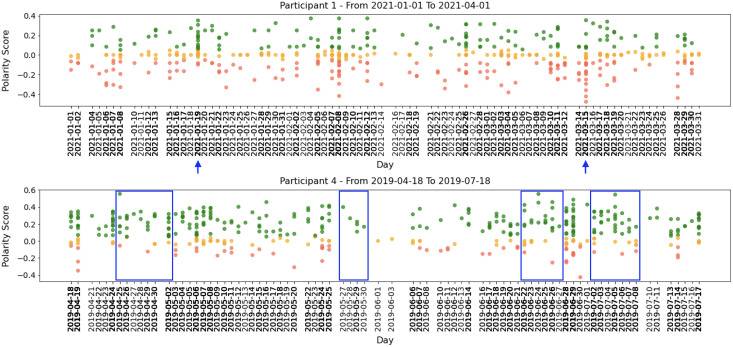
Examples of participants’ tweets polarities within 3 months sample.

All participants had periods of intense activity on Twitter interleaved with periods of less activity, and the sentiment polarity of developers’ posts changed considerably between positive and negative. Taking a closer look into small periods (days or weeks), we can identify short periods of polarity predominance, either positive or negative. Figure 5 shows examples of tweets for three months where the green points mean positive tweets, the orange points signify neutral, and the red ones represent negative tweets.

Globally, Figs. 4 and 5 show quite clearly that we can easily identify the overall polarity of the developers’ tweets in large periods, as well as the polarity of very specific points in time (*e.g*., a single day). For example, both participants shown in Fig. 5 had days with balanced positive and negative tweets. In contrast, some days (or short periods of a few days) show a clear predominance in negative or positive scores.

**Figure 6:**
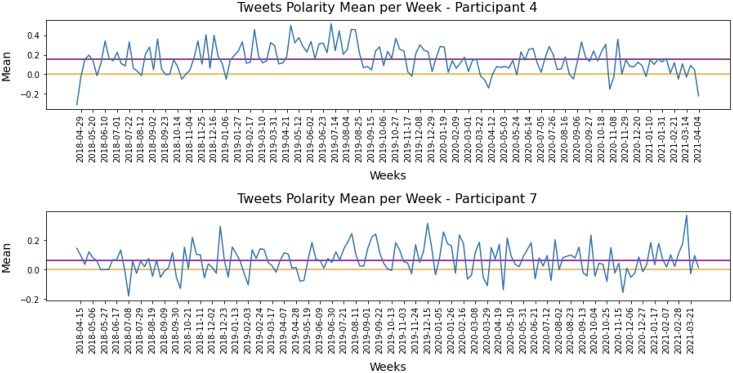
Polarity means grouped per week for Participant 4 and Participant 7.

The results allow us to establish a baseline for each developer’s sentiment polarity and detect variations from this baseline. This information could be used in future tools to assist software development teams in making decisions to improve the development process. For example, Fig. 6 illustrates the weekly sentiment means for P4 and P7. To improve readability, the figure only includes date labels every three weeks. The orange line represents a neutral sentiment score, while the purple line shows the overall sentiment mean for each participant, which serves as their baseline. Both P4 and P7 had a positive baseline throughout the study period.

It is interesting to note that P4 had clearly positive posts on Twitter during the period, with only a few negatives. The baseline for P4 was $0.15$, and most tweets were around the baseline. The baseline for P7 ( $0.07$) scored right above the threshold, and posts around neutral or weak-negatives posts could be considered normal activity. However, negative peaks for this participant may require attention.

### Personality traits correlation (RQ2)

The goal of this subsection is to investigate whether the personality traits profile of developers can be employed as an additional source to classify the polarity of posts.

Table 8 shows the detailed Big Five scores of the 16 participants and their percentage of tweets of each polarity. We used the Shapiro-Wilk test to assess the normality of the distribution of the tweets’ percentage variables (positive, neutral, and negative) and the personality trait factors variables. The results showed that the tweets’ percentage variables had a normal distribution, but the personality trait factors did not due to the limited number of participants.

We grouped scores into low and high categories using the codification from Table 8 to explore the correlations between personality traits and tweets’ polarities. Scores below 15 were grouped as low, while scores above 35 were grouped as high. For example, *Low Openness (LO)* group contains participants who scored less than 15 for *Openness*, while *High Openness (HO)* group includes participants who scored more than 35. Table 9 shows the group’s settings.

**Table 9:** Groups of participants according to Big Five scores.

Group	Participants	Quantity of participants
LO	–	0
HO	P1 to P3, P6, P9, P12	6
LC	–	0
HC	P1 to P3, P6, P8 to P15	12
LE	–	0
HE	P1, P6, P9, P15	4
LA	–	0
HA	–	0
LN	P5	1
HN	P4	1

The tweets’ percentage variables are continuous, and the personality trait factors are ordinal. Based on these assumptions, we used the non-parametric Spearman rank-order correlation coefficient to measure the correlation between developers’ sentiment polarities and their personality traits.

Some groups presented correlations with the percentage of tweets, while others did not. Table 10 displays the correlation matrix between the percentage of negative, neutral, and positive tweets and each personality trait for the HO group participants. The results indicate that *Openness*, *Conscientiousness*, and *Extraversion* may have a strong influence on the polarity of posts, particularly for positive and neutral ones. Three participants had a higher percentage of positive tweets, while the other three had a higher percentage of neutral tweets. None of them had a higher percentage of negative tweets than the other polarities. For example, P6 had 48.42% positive tweets, 28.51% neutral, and 23.07% negative tweets. The high scores in *Openness*, *Conscientiousness*, and *Extraversion* may have contributed to the positive posts. However, the low score on *Neuroticism* and moderate score on *Agreeableness* may have influenced the percentage of neutral and negative tweets.

**Table 10:** Correlation matrices showing the relationships between negative, neutral, and positive tweets and each personality trait for the analyzed groups.

Tweets (%)	Personality traits				
	Openness	Conscientiousness	Extraversion	Agreeableness	Neuroticism
HO group					
Negative	0.0857	−0.6179	−0.4928	0.0294	0.3479
Neutral	−0.3143	−0.7945	−0.7537	0.1471	0.4348
Positive	0.5429	0.7062	0.6377	−0.4708	−0.3479
HC group					
Negative	−0.0641	−0.0143	−0.3585	−0.4268	0.3620
Neutral	−0.2812	−0.6216*	−0.1301	0.0459	0.3128
Positive	0.1317	0.3858	0.0844	0.0247	−0.2425
HE group					
Negative	−0.8	−0.8	−0.7379	0.2	0.6
Neutral	−0.8	−0.8	−0.9487	−0.2	1.0
Positive	0.8	0.8	0.7379	−0.2	−0.6

**Note:**

* *p* < 0.05.

In the HO group, the correlation analysis suggests that if a developer posts more negative tweets than the group’s average, it may indicate a need for more attention from managers. This is because negative tweets have a weak correlation with the HO group’s expected polarity, and their presence may have a more negative impact than expected. Therefore, monitoring negative tweets can be an important indicator for identifying potential issues that require further attention from management.

The correlation analysis for the twelve participants of the HC group is presented in Table 10. Most of the group tended to post more neutral tweets (7/12), consistent with the typical behavior of individuals with high scores on *Conscientiousness*. These individuals are usually careful about their actions and decisions, paying attention to details and considering the consequences of their choices. However, within this group, the data also reveals that participants with lower scores on *Conscientiousness* tend to post more neutral tweets, which may appear contradictory despite the trend of people with high scores on this trait generally posting more neutral tweets. Nevertheless, it is worth noting that the polarities of posts are also influenced by the combinations of traits. For example, P13, P14, and P15 scored high on *Conscientiousness* (not as high as others) but had medium scores on *Openness* and *Neuroticism*, indicating that these traits may also influence the number of neutral tweets.

The correlation analysis suggests that developers in the HC group tend to post more neutral tweets, as there is a strong positive correlation between the percentage of neutral tweets and *Conscientiousness*. However, variations in scores for other personality traits may also affect the frequency of neutral or positive posts. Notably, participants in this group are less likely to post negative tweets, with only one participant (P10) having more negative tweets than the other polarities. Conversely, negative tweets can have a more negative impact than anticipated, as seen in the HO group. Thus, monitoring negative tweets can be a crucial indicator for identifying potential issues that require management’s attention.

The HE group consisted of four participants, two of whom had more positive tweets, and the other two had more neutral tweets. The participants with more positive tweets had the highest scores on *Extraversion*, as indicated by the strong positive correlation between this personality trait and positive tweets in Table 10. Individuals with high scores on *Extraversion* tend to be outgoing, energetic, and sociable, and they often have an optimistic outlook on life. These characteristics may explain why they tend to post more positive content on social media. The other two participants who posted more neutral tweets may be influenced by other personality traits, such as high scores on *Neuroticism*. As for HO and HC groups, monitoring negative tweets can be a crucial indicator for identifying potential issues that require management’s attention.

**Figure 7:**
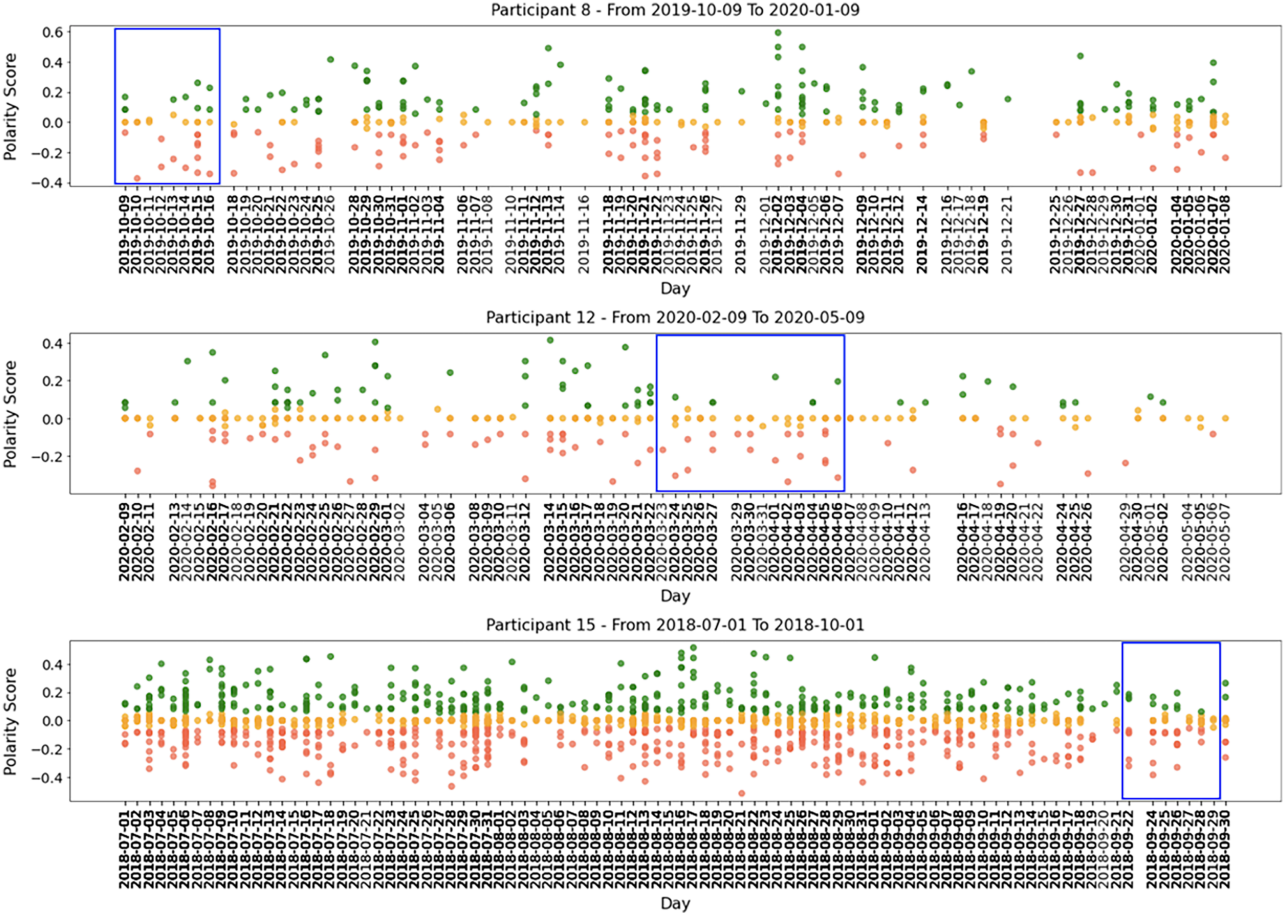
Examples of P8, P12, and P15 tweets’ polarities within 3 months period.

To exemplify our findings, we present examples of three participants. Participant 12 belongs to the HO group and obtained a Big Five score of *O = 36, C = 45, E = 31, A = 31, and N = 19*. As shown in Table 8, the high score in *Conscientiousness* may have contributed to a higher percentage of neutral tweets. Moreover, the combination of high *Conscientiousness* and *Openness*, as well as a medium score in *Extraversion*, may have influenced P12’s inclination to share positive tweets. Although this participant typically posts fewer negative tweets, medium scores in *Extraversion* and *Neuroticism* may contribute to these posts. For instance, the highlighted blue area in Fig. 7 (period 2020-02-09 to 2020-05-09) shows a few negative tweets.

We also analyze the social media post polarities of Participant 8, a member of the HC group, who scored *O = 29, C = 45, E = 34, A = 35, and N = 29* in the Big Five personality traits. The high score in *Conscientiousness* is consistent with the finding that members of the HC group tend to post more neutral tweets (Table 8). The medium scores in *Openness*, *Agreeableness*, and *Extraversion* may explain Participant 8’s tendency to share positive tweets. However, the medium score in *Neuroticism* suggests emotional instability and a tendency to express negative emotions, which is reflected in the highlighted blue area in Fig. 7 for the period 2019-10-09 to 2020-01-09.

Participant 15 scored *O = 29, C = 45, E = 36, A = 27, and N = 32* in the Big Five. As a member of the HC and HE group, P15 posted more neutral tweets (Table 8), as the score in *Extraversion* may have influenced the participant’s disposition to share positive tweets. However, the medium score in *Neuroticism* suggests a tendency to express negative emotions due to emotional instability. In fact, P15’s social media behavior was inconsistent at times. As shown in Fig. 7, the blue area in the chart from 2018-07-01 to 2018-10-01 indicates a prolonged period of negative sentiment polarity for consecutive days, which is unusual based on P15’s expected characteristics as inferred from the personality traits.

While the correlations observed between personality traits and emotional polarities in tweets can provide valuable insights into their classification, it is crucial to acknowledge that other factors may also impact these correlations. Human behavior is multifaceted, and factors such as the interaction between personality traits, life events, mood, situational context, and uncontrolled circumstances can all influence social media content, adding complexity to the correlation analysis. Additionally, it is important to note that correlation does not necessarily imply causation and may be more complex than a simple cause-and-effect relationship.

Our results provide insight into how personality traits may influence software developers through the correlation with tweets’ polarities, helping to interpret sentiment analysis results obtained through lexicons. Understanding a participant’s social media posts’ polarities and personality traits can enable software managers to anticipate and respond to deviations from their usual sentiment patterns. For instance, if P8, P12, and P15 suddenly display a significant increase in negative polarity, the manager may take proactive actions, such as assigning them to a low-complexity task or suggesting a break. These insights can inform strategic decisions that could improve the software development process.

### Working *vs* non-working tweets (RQ3)

We performed statistical analysis to verify a statistical difference between the polarities mean from tweets posted during the work period and posted outside the work period. We consider the Brazilian work time (from 9 am to 6 pm) a work period. Even with a large sample size ( $n \gt 5,\!000$), the data normality was confirmed by visual inspections of plots and a Shapiro-Wilk test. Thus, we performed a parametric z-test with a confidence level of $\alpha = 0.05$.

Table 11 shows the mean polarity score during the work period, outside the work period, p-value, and 95% confidence intervals for each participant. We found that for five participants (P2, P4, P5, P6, P12), the tests are statistically significant (*p* < 0.05), and thus we reject the null hypothesis ${H_0}$ for those participants, as highlighted in bold in Table 11. However, we cannot reject the null hypothesis ${H_0}$ for eleven participants (P1, P3, P7, P8, P9, P10, P11, P14, P15, P16), meaning that there was no statistical difference between the polarity means between tweets posted during the work period and tweets posted outside work period.

**Table 11:** Z-test statistic for working period *vs* non-working period tweets’ polarities.

Participant	Working period mean	Non-working period mean	*p*	95% CI
P1	−0.0131	−0.0150	0.6476	0.0020 [−0.0065 to 0.0104]
**P2**	**−0.0020**	**0.0068**	**0.0119**	**−0.0089 [−0.0158 to −0.0020]**
P3	0.0015	0.0025	0.8017	−0.0010 [−0.0086 to 0.0066]
**P4**	**0.0745**	**0.0988**	**0.0001**	**−0.0243 [−0.0369 to −0.0117]**
**P5**	**0.0399**	**0.0561**	**0.0001**	**−0.0162 [−0.0244 to −0.0079]**
**P6**	**0.0489**	**0.0640**	**0.0000**	**−0.0151 [−0.0218 to −0.0085]**
P7	0.0360	0.0510	0.0570	−0.0150 [−0.0305 to −0.0004]
P8	0.0359	0.0230	0.0617	0.0130 [−0.0006 to 0.0266]
P9	0.0606	0.0615	0.0806	−0.0009 [−0.0079 to 0.0061]
P10	−0.0094	−0.0076	0.6951	−0.0018 [−0.0111 to 0.0074]
P11	0.0447	0.0443	0.9316	0.0004 [−0.0098 to 0.0107]
**P12**	**−0.0188**	**0.0024**	**0.0007**	**−0.0230 [−0.0363 to −0.0096]**
P13	0.0008	−0.0011	0.6417	0.0020 [−0.0064 to 0.0103]
P14	−0.0079	−0.0069	0.8587	−0.0010 [−0.0123 to 0.0103]
P15	−0.0045	−0.0031	0.5385	−0.0014 [−0.0060 to 0.0031]
P16	0.0437	0.0502	0.1237	−0.0065 [−0.0149 to 0.0018]

**Note:**

Bold indicated change in mean.

Although we can not reject the null hypothesis for most participants, our results demonstrate that 31.25% (5/16) of participants provide valuable emotional information on social media outside of their work period. Thus, considering social media posts outside working time in sentiment analysis is highly indicated.

**Figure 8:**
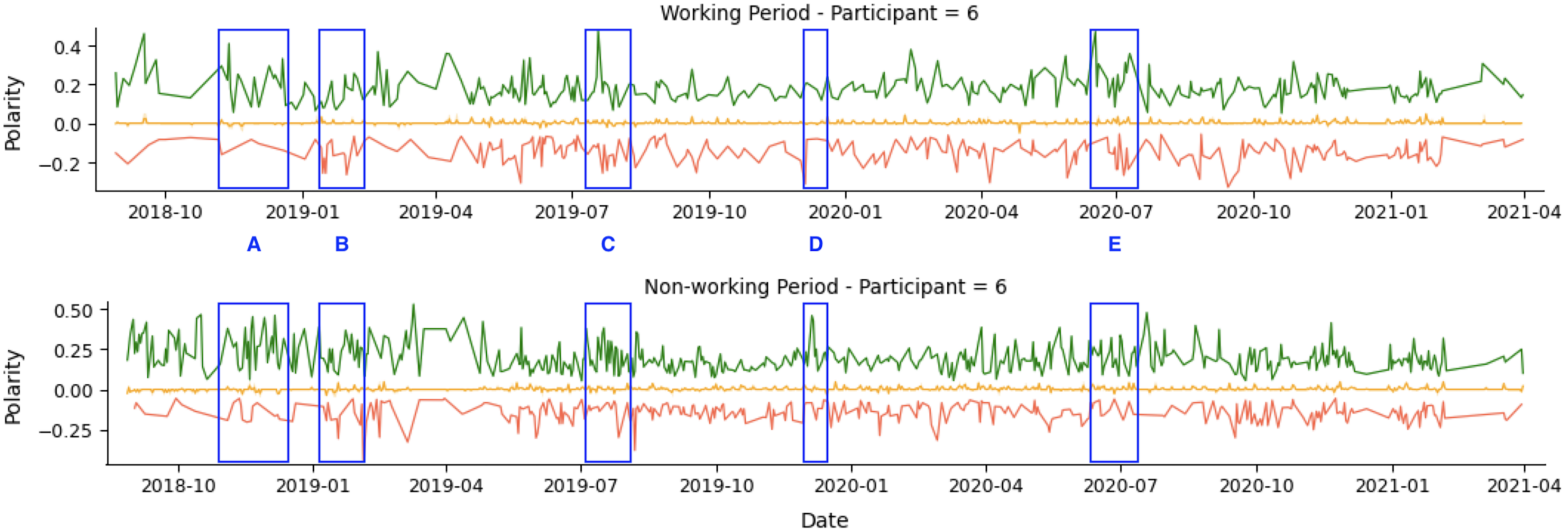
Working-time *vs* non-working-time tweets for Participant 6.

Figure 8 shows the tweets for Participant 6 during the study period divided into *working period* and *non-working period* groups. The figure shows five highlighted areas in both charts that indicate periods of changing polarity scores. In this figure, the green line represents positive tweets, the orange line signifies neutral, and the red represents negative tweets. The top chart shows the tweets during work periods, while the bottom exhibits the tweets outside work periods. The participant posted more positive tweets outside work hours in areas *A*, *B*, *C*, and *D*. The area *E* represents a balance in polarity but tends to be negative, indicating a possible happening during this period. Additionally, the area *C* shows a certain balance in polarity. However, it tends to be positive, which may have resulted from various factors, including a possible uncontrolled event in the participant’s life.

Figure 8 also shows a change in the score for positive polarity considering the mean of tweets’ polarities. During the working period, the participant posted tweets with a mean of $0.0489$, while outside the working period, the mean was $0.0640$. These changes in mean occurred to participants highlighted in bold in Table 11 may happen, in addition to those mentioned above, due to a post profile change or the personality traits.

The Big Five factor scores for Participant 6 (P6), as shown in Table 8, reveals medium-high scores on *Openness*, *Extraversion*, and a high score on *Conscientiousness*. Based on these scores, P6 tends to be creative, curious, imaginative, talkative, active, hard-working, well-organized, and punctual. This participant also scored medium-low on *Neuroticism*, meaning a calm, unemotional person. These characteristics may influence how this participant behaves on social media during the work period, such as posting professional stuff or texts with little or no embedded sentiment.

**Figure 9:**
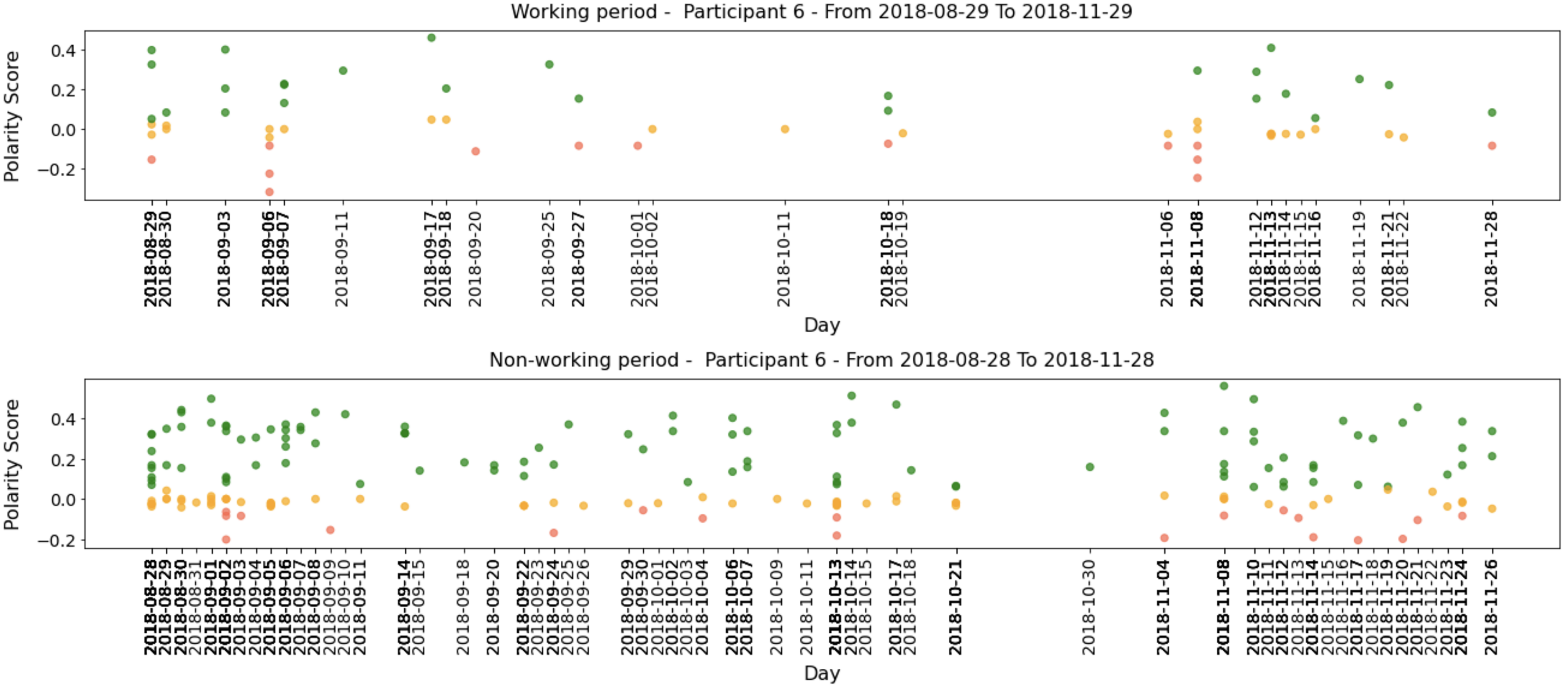
Three months period of working *vs* non-working tweets for Participant 6.

In Fig. 9, P6’s social media activity during the study period is divided into working and non-working periods. Green, orange, and red points represent positive, neutral, and negative tweets. The figure covers a 3-month interval (from 2018-08-28 to 2018-11-29). The top chart shows P6’s social media activity during work periods, while the bottom shows activity during non-work periods. It is evident from the figure that P6 posted more positive tweets during non-working periods, suggesting a change in behavior between work and leisure time. This may indicate that P6 has happy times, engaging in satisfying activities such as hobbies or sports, socializing with friends, or dating. However, as mentioned before, uncontrolled events in the participant’s life should be considered as external factors not captured in the data that may also influence the polarity of tweets.

### The approach in a software engineering environment

The proposed approach originated a web-based dashboard tool that displays emotional data about a development team (Silva et al., 2022). This dashboard provides a range of visualizations to support software managers in making decisions to improve the software development process, mainly focusing on software quality and developer productivity. The dashboard provides both team-level and individual-level feedback on sentiment over time, as well as displays each developer’s personality traits to improve data interpretation. The dashboard contains a set of useful information as follows:

- Displays a developer’s emotional state status by setting a threshold and visually highlighting developers that require attention from software managers. For instance, the dashboard shows the developers whose emotional polarities deviate significantly from their baseline or those with consistently negative sentiments. This could provide software managers with an at-a-glance view of which developers may need extra support or attention;
- Shows the team’s emotional sentiment polarity baseline, calculated as the mean of the software developers’ emotional polarities over a given period. This allows software managers to have a general understanding of the team’s overall emotional sentiment and gain valuable insight into the team’s emotional state. Software managers can then use this information to identify any correlations with team productivity or quality and take appropriate actions to improve it;
- Provides a detailed breakdown of tweets and their sentiment polarities over a given period. The tool highlights specific areas, divided into positive and negative. The negative area denotes a prolonged period of consecutive days or weeks, indicating a negative trend in emotional sentiment polarity for a particular developer. Conversely, the positive area indicates a prolonged period of consecutive days or weeks displaying a positive trend for that developer. This can give software managers a clear understanding of developers’ emotional polarities and take action accordingly.

With such a tool, software managers can make adjustments to the development process for a given day/period or a specific developer by reorganizing teams and development pairs to align tasks with the profiles and emotional polarities of developers. For instance, managers could suggest or reassign tasks based on the emotional polarity of developers inferred by our approach. Additionally, managers could assign lower-complexity tasks to a developer currently experiencing a negative emotional polarity, as these tasks are less likely to lead to errors. Conversely, developers in a stable polarity state could be given more complex tasks to work on that day. This approach can help to optimize the development process by aligning tasks with the capabilities and emotional polarities of the developers. Also, the tool could offer suggestions for actions that managers could take based on the provided insights.

Another practical implication of the use of our approach is that it could also benefit the quality assurance team by providing insights on testing needs for code produced by developers based on their emotional polarity. With an adaptation, the approach would understand the expected code quality from each developer based on their emotional polarity, guiding the quality assurance team on what to test carefully. This can help to ensure that the code produced by developers is of high quality and meets the standards expected by the company.

We anticipate the potential of our approach to improve a company’s continuous delivery process as well. Our approach could be integrated as a plugin into a company’s continuous delivery tool, allowing it to verify and only include source code/tasks developed by a specific set of developers under a particular emotional state or that have been thoroughly tested by the quality assurance team. This can help to ensure that the code being delivered is of high quality and has been developed by developers in a stable emotional state, reducing the risk of errors and promoting a more efficient delivery process.

The proposed approach and the envisioned set of tools could be applied to every software development company or a company with a software development department. However, it is important to note that our approach is not intended to diagnose mental and personality disorders. Diagnosis of mental issues should only be performed by qualified professionals such as trained psychologists and psychiatrists.

We designed our approach specifically targeting individuals (software developers in this study) who engage actively in social media and generate unique content. Social media is a common platform for developers to share technical and personal content, making it an ideal target for sentiment analysis. Twitter, known for its vast and diverse content, has been widely utilized in sentiment analysis research (Drus & Khalid, 2019). However, it is essential to highlight that our proposed approach is adaptable to incorporating other textual social media platforms. This flexibility allows us to accommodate various social media sources, catering to different contexts’ specific needs and preferences.

Implementing our approach in a software development environment may cause some inhibition among software developers, who may alter their behavior on social media. However, previous research has found that people tend to maintain consistent behavior based on their personality traits, and social media posts should reflect this behavior (Chen, Magdy & Wolters, 2020). Therefore, we expect developers would not drastically change their behavior or posting activity due to our non-intrusive approach, which ultimately aims to improve the software development process.

## Threats to validity

Although the results of the proposed approach of using social media posts to assess the polarity of sentiments of software developers during long periods of time are quite promising, it is important to discuss possible threats to the validity of the evaluation of our approach.

The reduced number of participants engaged in the study is a clear external limitation. Obviously, the 16 software developers and their tweets might not represent all software developers using Twitter (or another social media environment). However, we consider that the number of 16 software developers is enough to draw reliable conclusions, especially considering the very large number of posts considered (79,029) and the time span of 3 years considered for the social media activity of the participants.

The unbalance regarding the genre of the participants is an internal limitation for the validity of the results. Although we have tried to engage more female developers, the current strong gender unbalance in the software development market resulted in only six female developers, corresponding to 37.5% of the 16 participants. Although this is a limitation of the study, at least the set of participants used in the study can be seen as a correct representation of the software development market.

The limited number of participants in this study may have restricted the range of personality traits observed. Additionally, the fact that all participants scored high on *Conscientiousness* suggests a possible trend among professionals in this field, although further research with a larger sample size would be necessary to draw more robust conclusions. Examining the standard personality traits of software developers is not within the scope of this study.

Regarding generalisability, it is important to be cautious when interpreting the results of RQ2 and RQ3, as the small sample size of developers may limit the generalizability of the results. The limited number of participants and assessed personality traits may present challenges in drawing definitive conclusions.

The manual evaluation of tweet polarity by psychologists is a potential internal concern. However, a substantial number of tweets (560) were evaluated and divided equally among the participants. Despite some difficulties reported by the evaluators in classifying tweet polarity, the high agreement between self-classification by participants and evaluation by evaluators (accuracy of $0.865$, macro F1-Score of $0.855$, and Cohen’s Kappa Index of $0.710$) confirms the reliability of the ground truth polarity annotation. This reliability supports the validity of using Twitter as a data source and external evaluators to annotate a representative sample of posts for training supervised polarity classification methods. Using these evaluators has the advantage of avoiding asking software developers to classify the polarity of a sample of their posts, which would be difficult to apply in real software development scenarios.

## Conclusion and future work

The study of emotions and personality traits in professional contexts is a multidisciplinary effort that has gained significant attention. In this context, understanding factors shaping individual and organizational performance are important regardless of professional area.

Software development is a highly intensive human intellectual activity, and studying human factors and their impact on software engineering has gained increasing community attention. Sentiments, moods, and emotions clearly influence software developers’ motivation, with an unavoidable impact on the software development process. Available methods to assess psychological aspects of an individual, particularly the polarity of sentiments, such as self-assessed surveys, facial expressions analysis, and sensors attached to the user’s body impose a non-negligible degree of intrusiveness, which limits their actual utilization in real software development setups.

This article proposed using information from social media (*e.g*., developers’ posts on Twitter) as a new approach to assess the polarity of sentiments (*i.e*., negative or positive) of software developers. This approach can be used for long periods of time and does not cause any intrusiveness or disturbance in software developers, which means that it can be easily and immediately applied to real-world software development setups. However, although we contextualized this article in the field of software engineering, the proposed approach could be employed in other work areas.

Since the main question related to the proposed approach is whether it can be an accurate and reliable method to assess sentiment polarity or not, the first important goal of the present article is to evaluate the accuracy of the proposed approach through a comprehensive experimental study.

We collected a dataset of 79,029 tweets from 16 real software developers (experiment participants) during 36 months. Our first step was to evaluate the accuracy of the lexicons and ensembles of lexicons in a sample of 560 tweets. These tweets were evaluated manually by a group of three psychologists to establish a ground truth for the polarity of these 560 tweets. The evaluation produced by the psychologists was confirmed with a second manual evaluation performed by the authors of the tweets (*i.e*., the software developers that participated in the study), assuring that the polarity manually established for tweets really represents the ground truth for the evaluation of the lexicons.

Once we established the sample’s polarity, we evaluated the accuracy of five unsupervised sentiment analysis lexicons and ensembles of these lexicons in a total of 31 combinations of the lexicons. The best ensemble comprising SentiStrength, Sentilex-PT, and LIWC2015_PT (SS + SL + LI) lexicons achieved a macro F1-Score of $0.745$ on performing unsupervised sentiment classification, considering both negative and positive polarities and an accuracy of $0.768$. However, other lexicons, such as LIWC2015_PT (LI), achieved $0.0846$ of F1-Score for positive tweets and $0.772$ of accuracy, and Sentilex-PT (SL), achieved $0.686$ of F1-Score for negative tweets. The metrics reached by the ensemble SS + SL + LI indicate that the proposed approach can be applied in practice with Twitter as a valid data source, even using simple unsupervised sentiment classification methods.

After evaluating the accuracy of the 31 combinations of lexicons to identify the best alternative, we used the ensemble SS + SL + LI to evaluate the polarity of the entire set of 79,029 tweets. We showed that the proposed approach could provide an effective dashboard to display the sentiment polarity of software developers over time, giving project managers and team members useful information on the emotional status of the entire team at any moment.

The article also explores insights on the use of personality traits of software developers (evaluated using the Big Five model) to ponder the relevance of periods of negative or positive posts for each participant. The correlation analysis between personality traits and polarity of posts indicated that *Openness*, *Conscientiousness* and *Extraversion* were the most influential personality traits for positive and neutral tweets. In contrast, *Neuroticism* was the personality trait associated with negative tweets.

Five of our sixteen participants show a statistically different polarity when comparing the posts made during the work period and the ones posted outside the work period. This result reinforces the importance of an automated approach to performing sentiment analysis over data produced outside the work period instead of an approach restricted to the workplace.

In summary, our proposed approach uses available unsupervised lexicons to assess developers’ sentiment polarities from social media posts, providing a non-intrusive and easy method to gather such information. This approach can be used for various possibilities, including creating a dashboard that shows useful information on the emotional status of individual software team members. Our approach also enables fine-grained sentiment analysis by visualizing tweets and their polarities daily, allowing project managers to establish individual thresholds and trace baseline sentiment polarities for each developer. Such knowledge helps address significant changes in sentiment polarity as part of software team management.

The anonymized dataset used in the experimental study, including the answers to the demographic survey, the answers to the Big Five Inventory, the experiment protocol, the manual analysis from psychologists and participants, ethics committee documents, and all generated charts and data analysis are available for public access in the companion data (available at https://doi.org/10.5281/zenodo.7846996).

In future work, the authors plan to improve the tweet polarity classifier by including the personality traits scores obtained from the Big Five factor model to improve the objective sentiment analysis classification of tweets. We also plan to use the proposed approach in a real software development context to research possible ways to use the long-term monitoring of developers’ psychological state to provide specific team management recommendations to improve software quality and productivity.

## Additional Information and Declarations

### Data Availability

The following information was supplied regarding data availability: The source code is available at GitHub and Zenodo: https://github.com/leosilva/peerj_computer_science_2022/releases/tag/v1.0. Leo Silva. (2023). Source code for article “Using social media and personality traits to assess software developers’ emotional polarity”. Zenodo. https://doi.org/10.5281/zenodo.7864671. The data is available at Zenodo: Leo Silva, Marília Gurgel de Castro, Miriam Bernardino Silva, Milena Santos, Uirá Kulesza, Margarida Lima, & Henrique Madeira. (2023). Using social media and personality traits to assess software developers' emotional polarity [Data set]. Zenodo. https://doi.org/10.5281/zenodo.7846996.
